# Crystal structure, Hirshfeld surface analysis and computational studies of 5-[(prop-2-en-1-yl)sulfan­yl]-1-[2-(tri­fluoro­meth­yl)phen­yl]-1*H*-tetra­zole

**DOI:** 10.1107/S2056989019011459

**Published:** 2019-08-23

**Authors:** Yurii Slyvka, Evgeny Goreshnik, Nazariy Pokhodylo, Marian Mys‘kiv

**Affiliations:** a Faculty of Chemistry, Ivan Franko National University of Lviv, Kyryla i Mefodia Str, 6, 79005 L’viv, Ukraine; b Department of Inorganic Chemistry and Technology, Jožef Stefan Institute, Jamova 39, SI-1000 Ljubljana, Slovenia

**Keywords:** crystal structure, tetra­zole derivative, DFT calculation, Hirshfeld surface analysis

## Abstract

The title compound, C_11_H_9_F_3_N_4_S, was synthesized from 2-(tri­fluoro­meth­yl)aniline by a multi-step reaction. It crystallizes in the non-centrosymmetric space group *Pna*2_1_, with one mol­ecule in the asymmetric unit, and is constructed from a pair of aromatic rings [2-(tri­fluoro­meth­yl)phenyl and tetra­zole], which are twisted by 76.8 (1)° relative to each other because of significant steric hindrance of the tri­fluoro­methyl group at the *ortho* position of the benzene ring. In the crystal, very weak C—H⋯N and C—H⋯F hydrogen bonds and aromatic π–π stacking inter­actions link the mol­ecules into a three-dimensional network. To further analyse the inter­molecular inter­actions, a Hirshfeld surface analysis, as well as inter­action energy calculations, were performed.

## Abstract 2

The title compound was synthesized from 2-(tri­fluoro­meth­yl)aniline by a multi-step reaction and is constructed from a pair of aromatic rings [2-(tri­fluoro­meth­yl)phenyl and tetra­zole], which are turned by 76.8 (1)° relative to each other because of significant steric hindrance of the tri­fluoro­methyl group at the *ortho* position of the benzene ring·In the crystal, very weak C—H⋯N and C—H⋯F hydrogen bonds and aromatic π–π stacking inter­actions link the mol­ecules into a three-dimensional network.

## Chemical context   

Tetra­zoles are a well-known class of aromatic five-membered heterocycles, which have been investigated since the end of the 19th century. Their biological properties, including anti­viral, anti­cancer, anti-tuberculosis, anti­fungal and anti­oxidant activities have been shown by numerous studies (see, for example, Ostrovskii *et al.*, 2017 ▸). They also are increasingly regarded as efficient and selective inhibitors of enzymes governing the metabolic processes in the human body (Pegklidou *et al.*, 2010 ▸; Al-Hourani *et al.*, 2012 ▸; Aggarwal *et al.*, 2016 ▸).

Tetra­zoles are well established as suitable precursors for the construction of other nitro­gen-containing heterocycles such as pyrimidines (Shyyka *et al.*, 2018 ▸; Pokhodylo *et al.*, 2015 ▸), as well as being widely used as ligands in their own right to generate coordination compounds (Gaponik *et al.*, 2006 ▸; Aromí *et al.*, 2011 ▸). For example, allyl derivatives of 1*H*-tetra­zole-5-thiols have been used for the preparation of copper(I) π,σ-complexes possessing non-linear optical properties (Slyvka *et al.*, 2018 ▸, 2019 ▸). Among these, three copper(I) π,σ-coordination compounds, [Cu_2_(C_11_H_9_F_3_N_4_S)_2_(CF_3_SO_3_)_2_] (Slyvka, 2015 ▸), [Cu(C_11_H_9_F_3_N_4_S)_2_]BF_4_ and [Cu(C_11_H_9_F_3_N_4_S)(NH_2_SO_3_)(MeOH)] based on 5-[(prop-2-en-1-yl)sulfan­yl]-1-[2-(tri­fluoro­meth­yl)phen­yl]-1*H*-tetra­zole (I) (C_11_H_9_F_3_N_4_S) have been reported recently (Slyvka *et al.*, 2019 ▸). As part of our ongoing studies in this area, the synthesis and structure of the title compound, (I), are reported here.

## Structural commentary   

**Figure 1:**
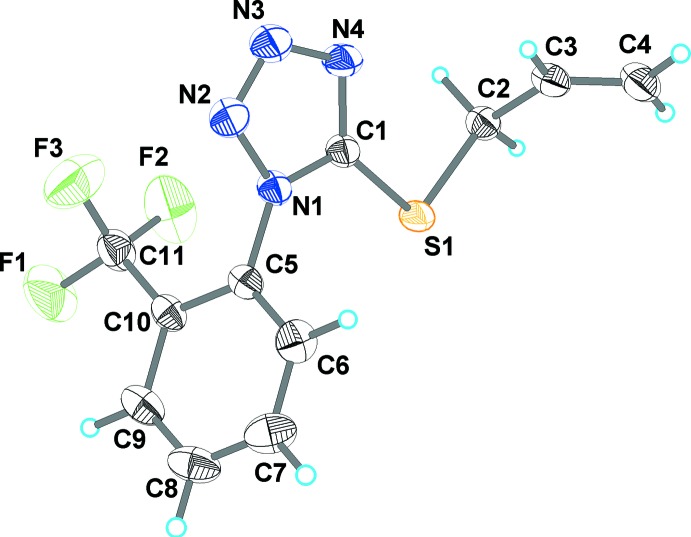
The mol­ecular structure of (I) with displacement ellipsoids drawn at the 50% probability level.

The title compound crystallizes in the non-centrosymmetric space group *Pna*2_1_, with one mol­ecule in the asymmetric unit. As shown in Fig. 1 ▸, it is constructed from two aromatic rings [2-(tri­fluoro­meth­yl)phenyl and tetra­zole rings], which are twisted relative to each other by 76.8 (1)° because of the significant steric hindrance of the tri­fluoro­methyl group attached to C10. This dihedral angle is comparable with the analogous parameter in the same ligand when it is π,σ-coordinated to a copper atom in [Cu(C_11_H_9_F_3_N_4_S)_2_]BF_4_ [dihedral angle = 78.0 (1)°] and [Cu(C_11_H_9_F_3_N_4_S)(NH_2_SO_3_)(MeOH)] [85.5 (1)°] (Slyvka *et al.*, 2019 ▸). The (prop-2-en-1-yl)sulfanyl group in (I) has an anti­clinal conformation relative to the C2—C3 bond and a synclinal conformation relative to the S1—C2 bond. The S1—C2—C3—C4 and C1— S1—C2—C3 torsion angles are 117.0 (3) and 75.0 (2)°, respectively.
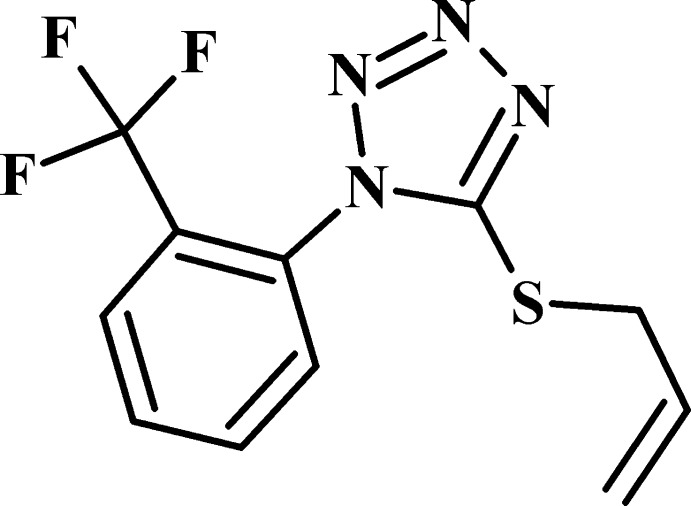


## Supra­molecular features   

**Figure 2:**
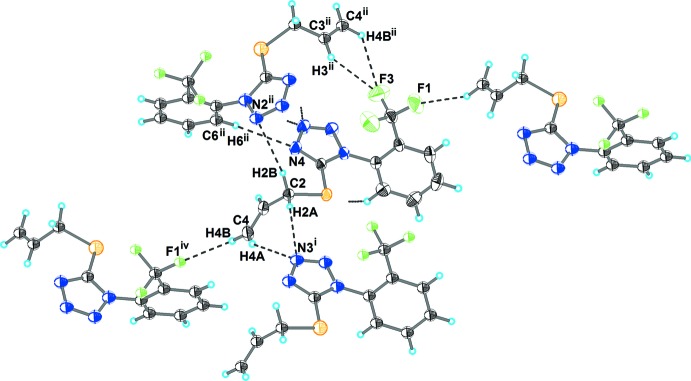
The hydrogen-bonding of mol­ecules in (I). Hydrogen bonds are shown as dashed lines. The symmetry codes are as in Table 1.

**Figure 3:**
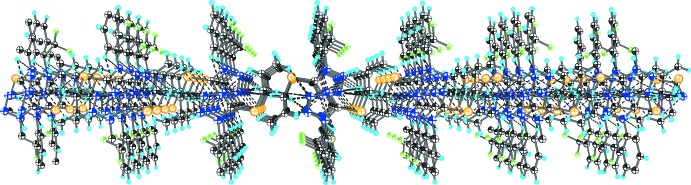
A C—H⋯N-bonded layer in the structure of compound (I).

**Figure 4:**
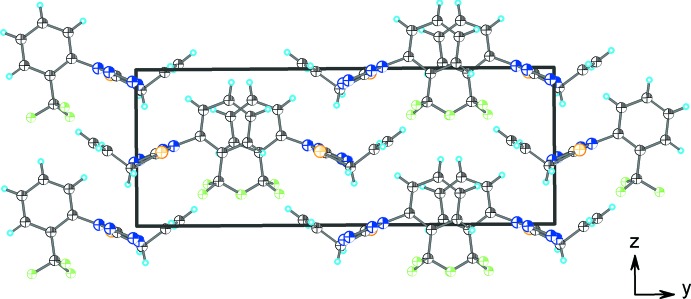
A view along the *a* axis of the crystal packing of the title compound.

As shown in Fig. 2 ▸ and listed in Table 1 ▸, the crystal structure of (I) features several weak inter­molecular inter­actions. The hydrogen atoms of the (prop-2-en-1-yl)sulfanyl group are involved in C—H⋯N bonding with the tetra­zole ring of an adjacent mol­ecule; these bonds link independent mol­ecules into layers (Fig. 3 ▸). The layers are inter­connected by C—H⋯F contacts into a three-dimensional network (Fig. 4 ▸).

## Hirshfeld surface analysis and computational study   

**Figure 5:**
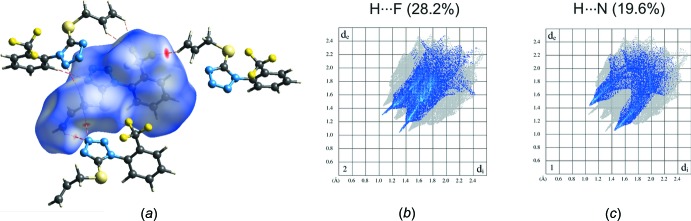
(*a*) Hirshfeld surface for mol­ecule of (I) mapped with *d*_norm_ over the range −0.15 to 1.2 showing C—H⋯N and C—H⋯F hydrogen-bonded contacts. Fingerprint plots for mol­ecule resolved into (*b*) F⋯H/H⋯F and (*c*) N⋯H/H⋯N contacts. Neighbouring mol­ecules associated with close contacts are also shown.

To further analyse the inter­molecular inter­actions between the mol­ecules of (I), Hirshfeld surface analysis through the mapping of the normalized contact distance (*d*_norm_) as well as calculation of the inter­action energies were performed using *CrystalExplorer* (Turner *et al.*, 2017 ▸; Spackman & Jayatilaka, 2009 ▸). The most prominent inter­actions among the allyl group H atoms and tetra­zole N atoms as well as among allylic H atoms and F atoms of neighbouring mol­ecules can be seen in the Hirshfeld surface plot as the red areas (Fig. 5 ▸ *a*). Fingerprint plots were produced to show the inter­molecular surface bond distances with the regions highlighted for C—H⋯F (Fig. 5 ▸ *b*) and C—H⋯N (Fig. 5 ▸ *c*) inter­actions. The contribution to the surface area for H⋯H contacts is 19.8%.

The inter­action energies in (I) were calculated using a dispersion-corrected CE-B3LYP/6-31G(d,p) quantum level of theory, as available in *CrystalExplorer*. The total inter­molecular energy is the sum of energies of four main components, *viz*. electrostatic, polarization, dispersion and exchange-repulsion factors of 1.057, 0.740, 0.871 and 0.618, respectively (Mackenzie *et al.*, 2017 ▸). The total calculated energy of the inter­molecular inter­actions of (I) is −115.9 kJ mol^−^. From Table 2 ▸, one can see the highest energy value (–36.2 kJ mol^−^) covers C—H⋯N and C—H⋯F inter­actions with the neighbouring mol­ecule generated by the symmetry code −*x* + 1, −*y* + 1, *z* − 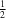. The inter­actions between the neighbouring 2-(tri­fluoro­meth­yl)phenyl rings stacked along [100] cover −25.7 kJ mol^−1^ and are mainly dispersive in nature.

## Database survey   

A survey of the Cambridge Structural Database (CSD version 5.39, last update August 2018; Groom *et al.*, 2016 ▸) confirmed that 1-aryl substituted 5-[(prop-2-en-1-yl)sulfan­yl]-1*H*-tetra­zoles are known only as ligands in the structures of copper(I) and silver(I) π-complexes. In the crystal structures of bis­[*μ* ^2^-*η* ^2^-5-(allyl­sulfan­yl)-1-phenyl-1*H*-tetra­zole]di­aqua­disilver bis(tetra­fluoro­borate) (refcode HAHTIV; Slyvka *et al.*, 2011 ▸), bis­{*μ*-*η* ^2^-1-phenyl-5-[(prop-2-en-1-yl)sulfan­yl]-1*H*-tetra­zole}di­aquadicopper bis­(tetra­fluoro­borate) (JAHCON; Slyvka *et al.*, 2010 ▸), bis­{*μ*-*η* ^2^-1-(4-chloro­phen­yl)-5-[(prop-2-en-1-yl)sulfan­yl]-1*H*-tetra­zole}di­aqua­dicopper bis­(tetra­fluoro­borate) ethanol solvate (JAHCUT; Slyvka *et al.*, 2010 ▸) and bis­{*μ*-5-[(prop-2-en-1-yl)sulfan­yl]-1-[2-(tri­fluoro­meth­yl)phen­yl]-1*H*-tetra­zole}bis­(tri­fluoro­methane­sulfonato)­dicopper (JADHII; Slyvka, 2015 ▸), the tetra­zole moieties are bonded to the metal ions through two heterocyclic nitro­gen atoms and the allylic C=C bond in the chelate-bridging mode. In *catena*-{(*μ*-sulfamato){*η* ^2^-1-(3,5-di­methyl­phen­yl)-5-[(prop-2-en-1-yl)sulfan­yl]-1*H*-tetra­zole}copper(I)} (ZEYRUT; Slyvka *et al.*, 2018 ▸) (VI), the organic mol­ecule is coordinated to the copper atom by the allylic C=C bond and the only tetra­zole nitro­gen atom. As a result of the presence of back-donation from an occupied 3*d* metal orbital to a low-lying empty π* orbital of the olefin, in all these compounds the double bond of the (prop-2-en-1-yl)sulfanyl group is slightly elongated to 1.35–1.38 Å, in comparison with noncoordinated olefin bond value. The other S-substituted 1-phenyl-1*H*-tetra­zole-5-thiol structures in the Cambridge Structural Database have different alkyl substit­uents, such as 2-naphthyl (TICRAY; Alves *et al.*, 1996 ▸), 1,7,7-tri­methylbi­cyclo­[2.2.1]hept-2-yl (GIJRAU; Bodrov *et al.*, 2013 ▸) and benzoyl (BAZVAA; Kim *et al.*, 2003 ▸).

## Synthesis and crystallization   

The title compound was synthesized from 2-(tri­fluoro­meth­yl)aniline by a multi-step reaction. Commercially available 2-(tri­fluoro­meth­yl)aniline (1.611 g, 0.010 mol) was dissolved in the minimum amount of benzene and treated with carbon di­sulfide (0.7 ml, 0.01 mol) and tri­ethyl­amine (1.4 ml, 0.010 mol). The solution was cooled to 273 K and left for 5 d. After complete precipitation of the tri­ethyl­ammonium di­thio­carbamate salt, the solution was filtered. The solid was washed with anhydrous ether and air-dried for about 10 min. The salt was then dissolved in about 7.5 ml of chloro­form, treated with 1.4 ml of tri­ethyl­amine and cooled to 273 K. To this solution was added ethyl chloro­formate (1.02 ml, 0.01 mol) dropwise over a 15 min period under intensive stirring. The resulting solution was stirred at 273 K for 10 min and allowed to warm to room temperature over 1 h. The chloro­form solution was washed with 3 *M* HCI and twice with water and dried over Na_2_SO_4_. The chloro­form was evaporated and the 1-iso­thio­cyanato-2-(tri­fluoro­meth­yl)benzene was distilled *in vacuo*.

The obtained iso­thio­cyanate (1.016 g, 5.0 mmol) was mixed with water (10 ml) and NaN_3_ (0.71 g, 0.011 mol) and refluxed under intensive stirring until the suspension disappeared. The solution was cooled to room temperature and washed with TBME. The water fraction was separated and acidified with 3 *M* HCl (Caution! During the acidification beware of toxic HN_3_ gas). The sediment of 1-[2-(tri­fluoro­meth­yl)phen­yl]-1*H*-tetra­zole-5-thiol was separated by filtration and used for alkyl­ation without further purification.

1-[2-(Tri­fluoro­meth­yl)phen­yl]-1*H*-tetra­zole-5-thiol (0.985g, 0.004 mol) was dissolved in a solution of KOH (0.22 g, 0.004 mol) in ethanol (10 ml). To the solution allyl bromide (0.43 ml, 0.005 mole) was added and the mixture was heated at 323 K for 1 h. The solvent was removed *in vacuo* and to the residue was added water (5 ml) and di­chloro­methane (10 ml). The di­chloro­methane was separated and removed to give the title compound. Colourless blocks of (I) were obtained by recrystallization from an ethanol solution, m.p. 336 K.

NMR ^1^H (400 MHz, DMSO-*d*_6_), δ, p.p.m. 8.03 (*d*, *J* = 7.3 Hz, 1H, H_Ph_-3), 7.98–7.88 (*m*, 2H, H_Ph_-4,5), 7.71 (*d*, *J* = 7.3 Hz, 1H, H_Ph_-6), 5.94 (*td*, *J* = 16.8, 7.2Hz, 1H, =CH), 5.36 (*d*, *J* = 16.8 Hz, 1H, =CH_2_), 5.18 (*d*, *J* = 9.9 Hz, 1H, =CH_2_), 3.98 (*d*, *J* = 6.9 Hz, 2H, CH_2_). Analysis calculated for C_11_H_9_F_3_N_4_S: C, 46.15; H, 3.17; N, 19.57; S, 11.20; found: C, 45.97; H, 3.04; N, 19.49; S, 11.27.

## Refinement   

Crystal data, data collection and structure refinement details are summarized in Table 3 ▸. H atoms were positioned geometrically and refined using riding model, with C—H = 0.95 or 0.99 Å and *U*_iso_(H) = 1.2*U*_eq_(C).

## Supplementary Material

Crystal structure: contains datablock(s) I. DOI: 10.1107/S2056989019011459/hb7842sup1.cif

Structure factors: contains datablock(s) I. DOI: 10.1107/S2056989019011459/hb7842Isup2.hkl

Supporting information file. DOI: 10.1107/S2056989019011459/hb7842Isup3.mol

Supporting information file. DOI: 10.1107/S2056989019011459/hb7842Isup4.cml

CCDC reference: 1947200

Additional supporting information:  crystallographic information; 3D view; checkCIF report

## Figures and Tables

**Table 1:** Hydrogen-bond geometry (Å, °)

*D*—H⋯*A*	*D*—H	H⋯*A*	*D*⋯*A*	*D*—H⋯*A*
C2—H2*A*⋯N3^i^	0.99	2.58	3.464 (3)	148
C2—H2*B*⋯N2^ii^	0.99	2.69	3.666 (4)	169
C3—H3⋯F3^iii^	0.95	2.71	3.351 (3)	125
C4—H4*A*⋯N3^i^	0.95	2.67	3.491 (3)	145
C4—H4*B*⋯F1^iv^	0.95	2.47	3.355 (3)	155
C6—H6⋯N4^iii^	0.95	2.76	3.601 (3)	148

Symmetry codes: (i) 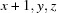; (ii) 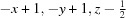; (iii) 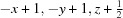; (iv) 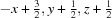.

**Table 2:** Inter­action energies (kJ mol^−1^) for selected close contacts in the crystal of (I)

Contact	*E*_electrostatic_	*E*_polarization_	*E*_dispersion_	*E*_exchange-repulsion_	*E*_total_	Symmetry operation
C4—H4*B*⋯F1	−1.4	−0.3	−5.2	5.7	−2.6	-*x* + 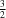 , *y* + 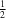 , *z* + 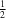
C4—H4*A*⋯N3/C2—H2*A*⋯N3	−12.0	−4.1	−13.4	16.6	−17.1	*x* + 1, *y*, *z*
C2—H2*B*⋯N2/C3—H3⋯F3/C4—H4*B*⋯F3	−14.6	−5.3	−31.0	16.4	−36.2	-*x* + 1, −*y* + 1, *z* − 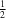
(CF_3_C_6_H_4_–)⋯(CF_3_C_6_H_4_–)	−5.0	−1.9	−31.8	14.1	−25.7	*x* − 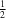 , −*y* + 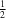 , *z*

**Table 3:** Experimental details

Crystal data	
Chemical formula	C_11_H_9_F_3_N_4_S
*M*_r_	286.28
Crystal system, space group	Orthorhombic, *P* *n* *a*2_1_
Temperature (K)	150
*a*, *b*, *c* (Å)	7.6595 (3), 20.9841 (7), 7.8641 (3)
*V* (Å^3^)	1263.98 (8)
*Z*	4
Radiation type	Mo *K*α
μ (mm^−1^)	0.28
Crystal size (mm)	0.35 × 0.24 × 0.15

Data collection	
Diffractometer	Rigaku Oxford Diffraction New Gemini, Dual, Cu at home/near, Atlas
Absorption correction	Analytical (*CrysAlis PRO*; Rigaku OD, 2018 ▸)
*T*_min_, *T*_max_	0.929, 0.969
No. of measured, independent and observed [*I* > 2σ(*I*)] reflections	28170, 3094, 2730
*R*_int_	0.059
(sin θ/λ)_max_ (Å^−1^)	0.680

Refinement	
*R*[*F* ^2^ > 2σ(*F* ^2^)], *wR*(*F* ^2^), *S*	0.035, 0.079, 1.07
No. of reflections	3094
No. of parameters	172
No. of restraints	1
H-atom treatment	H-atom parameters constrained
Δρ_max_, Δρ_min_ (e Å^−3^)	0.23, −0.22
Absolute structure	Flack *x* determined using 1094 quotients [(*I* ^+^)−(*I* ^−^)]/[(*I* ^+^)+(*I* ^−^)] (Parsons et al., 2013 ▸)
Absolute structure parameter	0.07 (4)

Computer programs: *CrysAlis PRO* (Rigaku OD, 2018 ▸), *SHELXT* (Sheldrick, 2015*a* ▸), *SHELXL* (Sheldrick, 2015*b* ▸) and *OLEX2* (Dolomanov *et al.*, 2009 ▸).

## supplementary crystallographic information

### Crystal data

**Table:**

C_11_H_9_F_3_N_4_S	*D*_x_ = 1.504 Mg m^−^^3^
*M**_r_* = 286.28	Mo *K*α radiation, λ = 0.71073 Å
Orthorhombic, *P**n**a*2_1_	Cell parameters from 9726 reflections
*a* = 7.6595 (3) Å	θ = 3.9–28.4°
*b* = 20.9841 (7) Å	µ = 0.28 mm^−^^1^
*c* = 7.8641 (3) Å	*T* = 150 K
*V* = 1263.98 (8) Å^3^	Block, colourless
*Z* = 4	0.35 × 0.24 × 0.15 mm
*F*(000) = 584	

### Data collection

**Table:**

Rigaku Oxford Diffraction New Gemini, Dual, Cu at home/near, Atlas diffractometer	2730 reflections with *I* > 2σ(*I*)
Detector resolution: 10.6426 pixels mm^-1^	*R*_int_ = 0.059
ω scans	θ_max_ = 28.9°, θ_min_ = 2.8°
Absorption correction: analytical (CrysAlis PRO; Rigaku OD, 2018)	*h* = −10→10
*T*_min_ = 0.929, *T*_max_ = 0.969	*k* = −28→28
28170 measured reflections	*l* = −10→10
3094 independent reflections	

### Refinement

**Table:**

Refinement on *F*^2^	Hydrogen site location: inferred from neighbouring sites
Least-squares matrix: full	H-atom parameters constrained
*R*[*F*^2^ > 2σ(*F*^2^)] = 0.035	*w* = 1/[σ^2^(*F*_o_^2^) + (0.0298*P*)^2^ + 0.4395*P*] where *P* = (*F*_o_^2^ + 2*F*_c_^2^)/3
*wR*(*F*^2^) = 0.079	(Δ/σ)_max_ < 0.001
*S* = 1.07	Δρ_max_ = 0.23 e Å^−^^3^
3094 reflections	Δρ_min_ = −0.22 e Å^−^^3^
172 parameters	Absolute structure: Flack *x* determined using 1094 quotients [(*I*^+^)-(*I*^-^)]/[(*I*^+^)+(*I*^-^)] (Parsons et al., 2013)
1 restraint	Absolute structure parameter: 0.07 (4)

### Special details

**Table:**

Geometry. All esds (except the esd in the dihedral angle between two l.s. planes) are estimated using the full covariance matrix. The cell esds are taken into account individually in the estimation of esds in distances, angles and torsion angles; correlations between esds in cell parameters are only used when they are defined by crystal symmetry. An approximate (isotropic) treatment of cell esds is used for estimating esds involving l.s. planes.
Refinement. 1. Fixed Uiso At 1.2 times of: All C(H) groups, All C(H,H) groups 2.a Secondary CH2 refined with riding coordinates: C2(H2A,H2B) 2.b Aromatic/amide H refined with riding coordinates: C3(H3), C6(H6), C7(H7), C8(H8), C9(H9) 2.c X=CH2 refined with riding coordinates: C4(H4A,H4B)

### Fractional atomic coordinates and isotropic or equivalent isotropic displacement parameters (Å^2^)

**Table:**

	*x*	*y*	*z*	*U*_iso_*/*U*_eq_	
S1	0.82933 (7)	0.43951 (3)	0.47114 (11)	0.02848 (15)	
F1	0.4353 (3)	0.24877 (9)	0.1989 (3)	0.0619 (6)	
F2	0.5490 (3)	0.34169 (11)	0.2020 (2)	0.0590 (6)	
F3	0.2811 (3)	0.32951 (12)	0.2643 (3)	0.0614 (6)	
N1	0.4878 (3)	0.41022 (10)	0.5110 (3)	0.0263 (5)	
N2	0.3257 (3)	0.43591 (10)	0.4866 (5)	0.0357 (6)	
N3	0.3518 (3)	0.49149 (11)	0.4245 (4)	0.0406 (7)	
N4	0.5253 (3)	0.50465 (11)	0.4058 (3)	0.0348 (6)	
C1	0.6066 (3)	0.45334 (10)	0.4616 (4)	0.0252 (5)	
C2	0.9068 (3)	0.51253 (12)	0.3701 (3)	0.0264 (5)	
H2A	1.030061	0.506647	0.335573	0.032*	
H2B	0.837482	0.520356	0.265975	0.032*	
C3	0.8943 (3)	0.56925 (10)	0.4825 (5)	0.0301 (5)	
H3	0.781849	0.582487	0.519235	0.036*	
C4	1.0299 (4)	0.60194 (13)	0.5336 (4)	0.0378 (7)	
H4A	1.143885	0.589750	0.498837	0.045*	
H4B	1.014415	0.637872	0.605561	0.045*	
C5	0.5129 (3)	0.34687 (11)	0.5739 (3)	0.0253 (5)	
C6	0.5647 (4)	0.33874 (16)	0.7405 (4)	0.0363 (7)	
H6	0.580710	0.374616	0.812478	0.044*	
C7	0.5933 (5)	0.27776 (17)	0.8017 (4)	0.0436 (8)	
H7	0.631875	0.271828	0.915363	0.052*	
C8	0.5662 (4)	0.22597 (15)	0.6992 (5)	0.0427 (8)	
H8	0.583610	0.184288	0.743224	0.051*	
C9	0.5136 (4)	0.23375 (13)	0.5317 (4)	0.0370 (7)	
H9	0.495146	0.197514	0.461602	0.044*	
C10	0.4879 (3)	0.29472 (11)	0.4665 (4)	0.0278 (5)	
C11	0.4394 (4)	0.30342 (14)	0.2845 (4)	0.0373 (7)	

### Atomic displacement parameters (Å^2^)

**Table:**

	*U*^11^	*U*^22^	*U*^33^	*U*^12^	*U*^13^	*U*^23^
S1	0.0220 (3)	0.0207 (2)	0.0428 (3)	0.0028 (2)	−0.0018 (3)	0.0025 (4)
F1	0.1032 (19)	0.0361 (10)	0.0464 (12)	−0.0050 (11)	−0.0153 (12)	−0.0163 (10)
F2	0.0844 (16)	0.0638 (14)	0.0287 (10)	−0.0314 (12)	0.0085 (10)	0.0003 (10)
F3	0.0620 (13)	0.0750 (15)	0.0472 (11)	0.0155 (11)	−0.0163 (10)	−0.0005 (11)
N1	0.0222 (10)	0.0226 (10)	0.0341 (14)	0.0019 (8)	0.0027 (9)	−0.0039 (9)
N2	0.0222 (9)	0.0327 (11)	0.0522 (16)	0.0065 (8)	0.0027 (12)	−0.0053 (13)
N3	0.0282 (12)	0.0328 (13)	0.0607 (19)	0.0074 (9)	−0.0022 (11)	−0.0004 (11)
N4	0.0269 (12)	0.0265 (11)	0.0511 (15)	0.0058 (9)	−0.0015 (11)	0.0045 (11)
C1	0.0223 (10)	0.0218 (10)	0.0317 (13)	0.0029 (8)	0.0014 (14)	−0.0030 (13)
C2	0.0260 (13)	0.0243 (13)	0.0290 (14)	−0.0003 (10)	0.0015 (11)	0.0011 (10)
C3	0.0306 (12)	0.0228 (11)	0.0368 (14)	0.0022 (9)	0.0041 (15)	0.0025 (14)
C4	0.0427 (16)	0.0251 (13)	0.0456 (18)	−0.0011 (12)	0.0008 (13)	−0.0017 (12)
C5	0.0232 (12)	0.0229 (12)	0.0297 (14)	−0.0001 (10)	0.0053 (11)	0.0001 (11)
C6	0.0404 (17)	0.0397 (17)	0.0288 (16)	0.0005 (14)	0.0036 (13)	−0.0043 (13)
C7	0.049 (2)	0.051 (2)	0.0311 (16)	0.0076 (15)	0.0049 (14)	0.0108 (14)
C8	0.0466 (18)	0.0338 (15)	0.0477 (19)	0.0052 (13)	0.0142 (15)	0.0146 (15)
C9	0.0415 (16)	0.0257 (13)	0.0440 (17)	−0.0033 (12)	0.0099 (13)	0.0025 (12)
C10	0.0279 (11)	0.0237 (11)	0.0318 (13)	−0.0034 (9)	0.0055 (14)	−0.0005 (13)
C11	0.0489 (19)	0.0294 (15)	0.0337 (16)	−0.0053 (13)	−0.0017 (13)	−0.0046 (12)

### Geometric parameters (Å, º)

**Table:**

S1—C1	1.732 (2)	C3—C4	1.308 (4)
S1—C2	1.825 (3)	C4—H4A	0.9500
F1—C11	1.330 (3)	C4—H4B	0.9500
F2—C11	1.331 (4)	C5—C6	1.380 (4)
F3—C11	1.340 (4)	C5—C10	1.396 (4)
N1—N2	1.367 (3)	C6—H6	0.9500
N1—C1	1.341 (3)	C6—C7	1.384 (5)
N1—C5	1.431 (3)	C7—H7	0.9500
N2—N3	1.280 (3)	C7—C8	1.369 (5)
N3—N4	1.365 (3)	C8—H8	0.9500
N4—C1	1.319 (3)	C8—C9	1.387 (5)
C2—H2A	0.9900	C9—H9	0.9500
C2—H2B	0.9900	C9—C10	1.392 (4)
C2—C3	1.486 (4)	C10—C11	1.490 (4)
C3—H3	0.9500		
			
C1—S1—C2	99.24 (12)	C6—C5—C10	121.1 (2)
N2—N1—C5	122.5 (2)	C10—C5—N1	120.0 (2)
C1—N1—N2	108.0 (2)	C5—C6—H6	120.3
C1—N1—C5	129.5 (2)	C5—C6—C7	119.3 (3)
N3—N2—N1	105.7 (2)	C7—C6—H6	120.3
N2—N3—N4	112.2 (2)	C6—C7—H7	119.8
C1—N4—N3	105.0 (2)	C8—C7—C6	120.4 (3)
N1—C1—S1	122.86 (17)	C8—C7—H7	119.8
N4—C1—S1	128.06 (19)	C7—C8—H8	119.7
N4—C1—N1	109.1 (2)	C7—C8—C9	120.6 (3)
S1—C2—H2A	109.0	C9—C8—H8	119.7
S1—C2—H2B	109.0	C8—C9—H9	120.0
H2A—C2—H2B	107.8	C8—C9—C10	120.0 (3)
C3—C2—S1	113.13 (19)	C10—C9—H9	120.0
C3—C2—H2A	109.0	C5—C10—C11	121.3 (2)
C3—C2—H2B	109.0	C9—C10—C5	118.5 (3)
C2—C3—H3	118.3	C9—C10—C11	120.1 (3)
C4—C3—C2	123.5 (2)	F1—C11—F2	106.8 (3)
C4—C3—H3	118.3	F1—C11—F3	105.7 (3)
C3—C4—H4A	120.0	F1—C11—C10	112.7 (2)
C3—C4—H4B	120.0	F2—C11—F3	105.5 (3)
H4A—C4—H4B	120.0	F2—C11—C10	112.6 (2)
C6—C5—N1	118.8 (2)	F3—C11—C10	112.9 (2)
			
S1—C2—C3—C4	117.0 (3)	C5—N1—N2—N3	−177.7 (2)
N1—N2—N3—N4	0.0 (4)	C5—N1—C1—S1	−1.9 (4)
N1—C5—C6—C7	−178.5 (3)	C5—N1—C1—N4	177.3 (2)
N1—C5—C10—C9	179.9 (2)	C5—C6—C7—C8	−1.6 (5)
N1—C5—C10—C11	1.5 (4)	C5—C10—C11—F1	174.8 (2)
N2—N1—C1—S1	−179.8 (3)	C5—C10—C11—F2	53.9 (4)
N2—N1—C1—N4	−0.6 (3)	C5—C10—C11—F3	−65.4 (3)
N2—N1—C5—C6	−104.5 (3)	C6—C5—C10—C9	1.1 (4)
N2—N1—C5—C10	76.6 (3)	C6—C5—C10—C11	−177.3 (3)
N2—N3—N4—C1	−0.3 (4)	C6—C7—C8—C9	1.4 (5)
N3—N4—C1—S1	179.7 (2)	C7—C8—C9—C10	0.0 (5)
N3—N4—C1—N1	0.6 (3)	C8—C9—C10—C5	−1.2 (4)
C1—S1—C2—C3	75.0 (2)	C8—C9—C10—C11	177.2 (3)
C1—N1—N2—N3	0.4 (4)	C9—C10—C11—F1	−3.6 (4)
C1—N1—C5—C6	77.8 (4)	C9—C10—C11—F2	−124.5 (3)
C1—N1—C5—C10	−101.1 (3)	C9—C10—C11—F3	116.2 (3)
C2—S1—C1—N1	175.3 (2)	C10—C5—C6—C7	0.3 (4)
C2—S1—C1—N4	−3.8 (3)		

### Hydrogen-bond geometry (Å, º)

**Table:**

*D*—H···*A*	*D*—H	H···*A*	*D*···*A*	*D*—H···*A*
C2—H2*A*···N3^i^	0.99	2.58	3.464 (3)	148
C2—H2*B*···N2^ii^	0.99	2.69	3.666 (4)	169
C3—H3···F3^iii^	0.95	2.71	3.351 (3)	125
C4—H4*A*···N3^i^	0.95	2.67	3.491 (3)	145
C4—H4*B*···F1^iv^	0.95	2.47	3.355 (3)	155
C6—H6···N4^iii^	0.95	2.76	3.601 (3)	148

Symmetry codes: (i) *x*+1, *y*, *z*; (ii) −*x*+1, −*y*+1, *z*−1/2; (iii) −*x*+1, −*y*+1, *z*+1/2; (iv) −*x*+3/2, *y*+1/2, *z*+1/2.
